# The Short-term Effects of a Cyberbullying Prevention Intervention for Parents of Middle School Students

**DOI:** 10.3390/ijerph14091038

**Published:** 2017-09-09

**Authors:** Anthony J. Roberto, Jen Eden, Douglas M. Deiss, Matthew W. Savage, Leslie Ramos-Salazar

**Affiliations:** 1 Hugh Downs School of Human Communication, Arizona State University, Tempe, AZ 85287, USA; 2 School of Communication and the Arts, Marist College, Poughkeepsie, NY 12538, USA; jen.eden@marist.edu; 3 Department of Communication and World Languages, Glendale Community College, Glendale, AZ 85302, USA; douglas.deiss@gccaz.edu; 4 School of Communication, San Diego State University, San Diego, CA 92182, USA; matthew.savage@sdsu.edu; 5 Computer Information & Decision Management, West Texas A&M University, Canyon, TX 79016, USA; lsalazar@mail.wtamu.edu

**Keywords:** cyberbullying prevention, susceptibility, behavioral intentions, parents

## Abstract

This study experimentally evaluated the short-term effects of the Arizona Attorney General’s cybersafety promotion presentation, a key component of which is cyberbullying prevention. Fifty-one parents of children attending a middle school in the southwestern United States participated in the study. Results reveal parents who viewed the presentation believed their children to be more susceptible to cyberbullying, and indicated that they were more likely to talk to their children about saving evidence, not retaliating, and telling an adult compared to parents who had not viewed the presentation. The theoretical and practical implications of these results are discussed.

## 1. Introduction

*Cyberbullying* is “the deliberate and repeated misuse of communication technology by an individual or group to threaten or harm others” [1] (p. 199). Cyberbullying has been a persistent public health concern for adolescents for at least two decades. This issue urged scholars to conduct applied research to support the progress and evaluation of effective cyberbullying prevention programs. As one step toward addressing this important gap in the literature, a pilot study was conducted to experimentally evaluate the short-term effects of the Arizona Attorney General’s cybersafety promotion presentation on the parents of middle school students. As will be discussed in more detail below, the primary purpose of this manuscript is to evaluate one specific intervention strategy that emphasizes how parents can encourage their children to respond once they have been cyberbullied. 

Given the evaluated intervention was designed and implemented by the Arizona Attorney General’s office, a brief overview of U.S. and Arizona anti-bullying and anti-cyberbullying laws seems warranted. Hinduja and Patchin [2] provide detailed information on anti-bullying and anti-cyberbullying laws for all 50 U.S. states. Specifically, anti-bullying legislation has been passed in all 50 U.S. states, and anti-cyberbullying legislation has been passed in 23 states. For example, in the state of Arizona (where the intervention being reported on here was designed, implemented, and evaluated), the anti-bullying laws both (1) “include harassment, bullying, and intimidation with the use of electronic technology,” and (2) “requires school district governing boards to adopt and enforce procedures that prohibit the harassment, bullying and intimidation of pupils on school grounds, school property, school buses, school bus stops and at school sponsored events and activities” [2] (p. 2).

Mason [3] notes that it is vital that schools work with families to educate adolescents about cyberbullying and its prevention. To illustrate, evidence suggests that working with parents is an important part of any well-rounded strategy to address cyberbullying that has the potential to be effective. Farrington and Ttofi [4] conducted a meta-analysis of the qualities and effects of 44 school-based bullying programs and how to openly discuss cyberbullying with their children, when to meet with school administrators, when and how to work with a cyberbully’s parents, or how to request help with an Internet service provider or law enforcement agency to address incidents. As a result of their systemic review of cyberbullying scholarship, Aboujaoude, Savage, Starcevic, and Salame [5] argued that a comprehensive approach to combat cyberbullying among adolescents is likely to succeed by including educational media campaigns, school-based programs, parental oversight and involvement, legislative action, and screening and evidence-based interventions by health care providers. A tenet of educational scholarship determined that parental involvement was associated with a significant reduction in victimization and perpetration. When it comes to cyberbullying, Moreno [6] points out that parents can be taught that schools must work closely with parents to combat complex problematic behavior like cyberbullying. 

Further, the international and national literature also highlights the role of parental involvement in cyberbullying prevention. An EU Kids Online survey, for instance, found that the majority of parents (74%) manage their children’s Internet use by talking directly to the children [7]. Another UK study found that parents adopt a variety of mediation strategies (e.g., technological restrictions) to prevent online risks; however, the restriction of peer-to-peer online interactions was shown to be the most effective [8]. To prevent cyberbullying in the UK, Cowie [9] recommends that interventions need to train the children and their parents about Internet safety and counteractive technological tools. Yet, in Finland where 20% to 30% of youth are cyberbullied, Salmivalli and Pöyhönen [10] report that parental involvement in cyberbullying prevention interventions is needed because parents’ supervision of their children’s Internet use is lax, with 44% of Finnish youth reporting no parental limitations, 41% reporting infrequent limitations, and 8% reporting firm limitations. 

In preventing cyberbullying in the U.S., Willard [11] suggests intervention strategies for educating parents such as installing monitoring software to monitor their child’s Internet activities, restricting their child’s computer access, and helping cyberbullying victims file a complaint. An American study by Hinduja and Patchin [12] also found that parents who interacted with their children about the unacceptability of cyberbullying served as a valuable social influence in preventing cyberbullying behaviors in both middle and high school students. Additionally, Hinduja and Patchin [13] recommend parental involvement strategies such as using open communication, going online with their children, developing practices for safe Internet use, and monitoring their online activities. Thus, the literature suggests that parental involvement is invaluable in addressing cyberbullying issues.

Three common recommendations made by experts in various related fields to help identify and prevent cyberbullying include (1) saving evidence, (2) not retaliating, and (3) telling a trusted adult or authority figure [14]. A key goal of the presentation being evaluated in this study was to get parents to talk to their children about all three of these recommended responses. Regarding saving evidence, a key difference between traditional bullying and cyberbullying is that evidence of traditional bullying is difficult to collect and preserve, whereas evidence of cyberbullying is relatively easy to collect and preserve [15,16]. So, saving evidence is recommended because it can be used to both investigate and intervene in cyberbullying incidents. However, while the ability to save evidence is a behavior that most cyberbullying victims report knowing how to do [17], the advancement of technologies such as Snapchat pose problems as the messages self-destruct once they have been viewed [18]. This becomes particularly problematic for law enforcement as potential criminal evidence is deleted, not just from the sender and receiver’s devices, but also from Snapchat’s servers as well [19]. Not retaliating is recommended because it can stop the incident from spiraling out of control, and just as importantly it serves to prevent the victim from potentially becoming a cyberbully themselves [16,20,21,22]. Finally, notifying a trusted adult or authority figure is recommended because it can help cyberbully victims better handle the situational stress of victimization. For example, a large body of literature has consistently found a positive relationship between social support and a variety of relationship, health, and psychological [23]. Unfortunately, research suggests that over 90% of children do not tell an adult when they are cyberbullied [17,24,25]. 

## 2. The Extended Parallel Process Model

This study uses the extended parallel process model (EPPM) [26] as an investigatory framework. The EPPM talks about the importance of four key input variables in changing attitudes, intentions, and behavior: (1) susceptibility (i.e., the likelihood that a threat will occur), (2) severity (i.e., how harmful a potential threat is), (3) response efficacy (i.e., a belief that a suggested response will be effective), and (4) self-efficacy (i.e., a belief that one is able to enact a recommended response). Given the content of the presentation and the short-term nature of this study, this investigation focuses on behavioral intentions, or what a person plans or intends to do [27]. In sum, the EPPM was chosen to guide this study since the presentation clearly included both an important input variable (i.e., perceived susceptibility) and outcome variable (i.e., intentions) from this model. Given this, in tandem with the information provided above about recommended responses to cyberbullying provided earlier, the following hypotheses are advanced:

**Hypothesis** **1** **(H_1_).** *Parents exposed to the experimental message will report their children are more susceptible to cyberbullying than those who do not view the presentation.*

**Hypothesis** **2a** **(H_2a_).** *Parents exposed to the experimental message will demonstrate greater intentions to talk to their children about saving evidence.*

**Hypothesis** **2b** **(H_2b_).** *Parents exposed to the experimental message will demonstrate greater intentions to talk to their children about not retaliating.*

**Hypothesis** **2c** **(H_2c_).** *Parents exposed to the experimental message will demonstrate greater intentions to talk to their children about telling a trusted adult than those in the control group.*

## 3. Method

### 3.1. Participants

All parents who attended a presentation at a middle school in a large southwestern city agreed to take part in the study. In total, 51 parents of middle-school children participated. A majority of these parents were women (84.3%; men = 15.7%). Parents reported their age as being 35 or younger (23.5%), 36–40 years old (9.8%), 41–45 years old (29.4%), 46–50 years old (25.5%), 51 or older (11.89%). Ninety-two percent were the biological parents of the student at the middle school, 4% were step-parents, and 4% were another relative (i.e., older sister/brother, aunt/uncle, grandmother/grandfather, etc.). Parents responded to items concerning their middle school girls (66.7%) and boys (33.3%) who were in the 5th grade (21.6%), 6th grade (25.5%), 7th grade (37.3%), or 8th grade (15.7%). In terms of race/ethnicity, the sample was white (76.5%), black (11.8%), Hispanic, (7.8%) and American Indian (3.9%).

### 3.2. Design and Procedures

This pilot study evaluates the short-term effects of the Arizona Attorney General’s cyberbullying prevention presentation on parents of middle school students using a separate-sample pretest-posttest design with random assignment to conditions [28,29]. That is, researchers randomly assigned parents to one of two groups: (1) an experimental group that viewed the presentation before completing the survey measuring the dependent variables, and (2) a control group that completed the survey measuring the dependent variables before viewing the presentation. The researchers worked with school officials in order to provide childcare, food and non-alcoholic beverages for the parents during the presentation. All participating parents were also entered into a drawing to receive a Starbucks gift card. The presentation lasted approximately one hour, and the survey took approximately 15 min to complete. 

### 3.3. Intervention

Since the researchers were evaluating an already existing presentation, the presentation was not explicitly guided by a communication theory. However, after viewing the presentation and having in-depth discussions with one of its creators, it was clear that some of the key goals were to increase parents’ beliefs that their children were susceptible to cyberbullying, and to get parents to talk to their children about saving evidence, not retaliating, and telling a trusted adult if they were cyberbullied. It should be noted that the parent presentation is actually an adaptation of a presentation that was originally created for middle school students themselves. The three key differences were that this one (1) used parents rather than students as the point of reference, (2) focused on what parents needed to do to help protect their children, and (3) had a greater focus on cyberbullying, as opposed to cybersafety in general. The authors were also involved in the evaluation of the effects of the intervention on middle school students [20].

With this in mind, the parent version of the presentation typically runs between 45 and 55 min. Examples of how perceived susceptibility to cyberbullying was addressed included defining cyberbullying, providing several anecdotal examples and news stories that demonstrated what cyberbullying was and the negative impacts it could have, reviewing several news stories of adolescents who had been arrested for cyberbullying, and reviewing Arizona’s cyberbullying prevention laws. The presentation concluded by reviewing ways parents could talk about cyberbullying in general with their children, as well as strategies for discussing the importance of saving evidence, not retaliating, and telling a trusted adult or authority figure if they were ever a victim of cyberbullying. 

### 3.4. Instrumentation

All measures were adapted from Ajzen and Fishbein [27] and/or Witte, Cameron, McKeon, and Berkowitz [30] and were assessed using five-point Likert scales. The general instructions for the survey read:

Sometimes a person or group of people (that is, friends, classmates, or maybe people we don’t even know) use cell phones or the Internet to *repeatedly* send or post messages in order to intentionally threaten or hurt people, make them feel bad, or to embarrass people in front of others in an *unfriendly way*.
For example, a person might send several messages directly to someone using a cell phone or email. Or, a person might post photos or messages about someone in places other people can see like on a Website.
Please keep this information in mind when answering the questions in this section of the survey.

These instructions were adapted from Roberto and Eden’s [1] definition of cyberbullying, and immediately followed by the measures of the dependent variables under investigation.

*Perceived susceptibility* (i.e., “It is possible that someone else will use a cell phone or the Internet to hurt or embarrass my child in the future”) was measured using three items (α = 0.81, *M* item score = 4.29, *SD* = 0.83). *Behavioral intentions* regarding talking to their children about saving evidence, not retaliating, and telling an adult were each measured with a single item (i.e., “I intend to talk to my child about *saving evidence*/*not retaliating*/*telling an adult* if someone else uses a cell phone or the Internet to hurt or embarrass them in the future”). 

The survey also asked a variety of questions about their child’s access to various communication technologies (i.e., e-mail, cell phones, personal computers, and the Internet), as well as their child’s past experiences with cyberbullying victimization and perpetration (i.e., “In the past year, did [anyone else/your child] ever use a cell phone or the Internet to send or post messages or images to hurt or embarrass [your child/anyone else] in an unfriendly way?”).

## 4. Results

### 4.1. Technology Access and Cyberbullying Prevalence 

A majority of parents reported that their children had access to a wide variety of their own communication technologies such as personal e-mail accounts (66.7%), cell phones (58.8%), personal computers (88.2%), and the Internet (82.4%). Further, 37.3% of parents reported that their children had their own social networking site such as Facebook. In terms of cyberbullying, approximately 61% of parents reported that their child had *not* been a victim, 22% reported that their child had been a victim, 16% of parents did not know. Further, 75% of parents reported that their child had *not* been a perpetrator, 12% reported that their child had been a perpetrator, and 14% parents did not know. 

### 4.2. Cyberbullying Prevention

H_1_ predicted that parents exposed to the experimental message would perceive their children as more susceptible to cyberbullying than those in the control group. As indicated by the means, standard deviations, and *t*-tests reported in Table 1, the experimental group reported significantly higher perceptions of susceptibility than the control group. Thus, H_1_ was supported. 

H_2a_ predicted that parents exposed to the experimental message would demonstrate greater intentions to talk to their children about saving evidence when cyberbullied than those in the control group. As reported in Table 1, the experimental group had significantly higher intentions than the control group for this variable. Thus, H_2__a_ was supported. H_2__b_ predicted that parents exposed to the experimental message would demonstrate greater intentions to talk to their children about not retaliating when cyberbullied than those in the control group. As can be seen in the results from Table 1, the experimental group did, in fact, report significantly higher intentions than the control group for this variable. Thus, H_2__b_ was supported. Finally, H_2__c_ predicted that parents exposed to the experimental message would demonstrate greater intentions to talk to their children about telling a trusted adult when cyberbullied than those in the control group for this variable. Thus, H_2__c_ was supported.

## 5. Discussion

This study investigated the short-term effects of the Arizona Attorney General’s cybersafety promotion presentation on parents of middle school students on two practically and theoretically important dependent variables: perceived susceptibility and behavioral intentions. Previous research on cyberbullying has focused primarily on adolescents’ first hand experiences with cyberbullying victimization and perpetration [15,16,17,20,24,31]. To our knowledge this is one of only a few studies that focused on parents [25], and is the first of its kind to experimentally test a cyberbullying prevention intervention targeting parents of middle school students. 

In this study, significant differences were found between parents in the experimental and control groups for all cyberbullying outcomes. Specifically, parents who viewed the presentation were more likely to believe it was possible that someone else might cyberbully their child in the future (i.e., they demonstrated greater perceived susceptibility). Further, and in comparison to the control group, parents in the experimental group indicated they were more likely to talk to their child about the importance of saving evidence, not retaliating, and telling a trusted adult if they ever experienced cyberbullying. 

These results are consistent with previous EPPM research, which suggests that increases in perceived threat (in this case susceptibility) should lead to increases in intentions [32]. This supposition is further supported by the fact that the correlations between susceptibility and the three measured behavioral intentions ranged from 0.39 to 0.55 (*p* < 0.002) in this study. This is substantially larger than the reported relationship between susceptibility and intentions in the Witte and Allen meta-analysis (*r* = 0.17, *p* < 0.05). Unfortunately, a number or practical concerns prevented us from focusing on other EPPM variables in this study. First, we were evaluating an existing intervention. Moreover, while this intervention included components that fit well conceptually with the EPPM, it did not include strong messages regarding all of the components of this model. Second, we were asked to keep the survey as short as possible to minimize the demands placed on the parents taking part in the study. In tandem, this lead to the choice to measure just the variables that were a key focus of the intervention being evaluated. In the future, the researchers hope to develop their own intervention to have more control over both the content and the measured evaluation outcomes.

## 6. Strengths and Limitations

This study benefits from a number of notable strengths. For example, it was conducted in a realistic and natural field setting (making it higher in external/ecological validity), and included parents of middle school students as a sample. Further, it used a quasi-experimental design that protects against most threats to internal validity. In fact, the two key internal validity threats to this experimental design according to Campbell and Stanley [28] are history (which can be ruled out since participants were sequestered) and maturation (which can be ruled out since the study took place over such a short period of time). Finally, this study focuses on an important topic (cyberbullying prevention) that has important practical implications (i.e., to our knowledge this is the first time a cyberbullying prevention intervention targeting parents has been experimentally tested). 

As with any investigation, some limitations are also worth noting. First, as indicated in the title, this study focused on short-term changes in perceived susceptibility and intentions. Future research would benefit from a longitudinal study that could assess the long-term effects on these and other variables. Second, and on a related note, this study focused on intentions rather than actual behavior. And, while we acknowledge that behavior is the gold standard when assessing the effects of any intervention, we are encouraged by the numerous meta-analyses that have consistently found medium to large effect sizes between intentions and behavior in a wide variety of contexts [33,34,35,36]. Finally, 51 parents of middle school students from one school participated in this study. And, while larger sample sizes are generally desirable, it is notable that we were still able to find some relatively large effects on the variables under investigation even with this sample size (i.e., the effect sizes ranged from 0.63 to 0.79, which should be interpreted as medium to large [37]. 

## 7. Conclusions

This study evaluated the short-term effects of the Arizona Attorney General’s cybersafety promotion presentation among parents of middle school students using a quasi-experimental design. As hypothesized, parents who viewed the presentation perceived their children to be more susceptible to cyberbullying, and indicated greater intentions to talk to their children about saving evidence, not retaliating, and telling an adult if they became the victims of cyberbullying. While this investigation suggests that even a short intervention can have a meaningful impact on several theoretically important variables, additional research in this area is still needed. 

## Figures and Tables

**Table 1:** Means (and standard deviations) for all hypotheses.

Hypothesis	Experimental Group *n* = 26 *M* (*SD*)	Control Group *n* = 25 *M* (*SD*)	*t*
H_1_: Susceptibility	4.59 (0.69)	3.99 (0.86)	*t* (49) = 2.76, *p* = 0.01, *d* = 0.79
H_2a_: Save Evidence	4.81 (0.63)	4.20 (1.26)	*t* (49) = 2.19, *p* = 0.03, *d* = 0.63
H_2b_: Not Retaliate	4.96 (0.20)	4.32 (1.22)	*t* (49) = 2.66, p = 0.01, *d* = 0.76
H_2c_: Tell Adult	5.00 (0.00)	4.56 (1.00)	*t* (49) = 2.24, *p* = 0.03, *d* = 0.64

Note: Because the presentation dealt separately with intentions to talk to children about saving evidence, not retaliating, and telling an adult, we chose to analyze the data for these individual items separately. However, it is also possible to combine these items into an overall behavioral intentions scale and analyze them together. When doing so the alpha for this three-item scale is 0.88, the mean for the experimental group is 4.92 (*SD* = 0.23), the mean for the control group is 4.36 (*SD* = 1.05), and *t* (49) = 2.65, *p* = 0.01, *d* = 0.77. H_1_: Hypothesis 1; H_2a_: Hypothesis 2a; H_2b_: Hypothesis 2b. H_2c_: Hypothesis 2c.
